# Motivation to change drinking behavior: the differences between alcohol users from an outpatient gastroenterology clinic and a specialist alcohol treatment service

**DOI:** 10.1590/S1516-31802005000500005

**Published:** 2005-09-01

**Authors:** Neliana Buzi Figlie, John Dunn, Luis Cláudio Santoro Gomes, Janaina Turisco, Roberta Payá, Ronaldo Laranjeira

**Keywords:** Alcoholism, Motivation, Gastroenterology, Outpatients, Needs assessment, Alcoolismo, Motivação, Gastroenterologia, Pacientes ambulatoriais, Determinação de necessidades de cuidados de saúde

## Abstract

**CONTEXT AND OBJECTIVE:**

For some patients who have developed significant alcohol-related physical disease, total abstinence from alcohol may offer the best chance of survival. The aim of this study was to investigate motivation for treatment in two groups of alcohol users: outpatients from the gastroenterology clinic and outpatients from the specialist alcohol treatment service.

**DESIGN AND SETTING:**

Cross-sectional study, at a federally funded public teaching hospital.

**METHODS:**

The sample studied was 151 outpatients from the gastroenterology clinic and 175 from the specialist alcohol treatment service. The interview was conducted in the outpatient clinics at the first appointment, and consisted of demographic questions and scales for measuring quality of life, alcohol dependence, pattern of alcohol, motivation for treatment and consequences of alcohol consumption.

**RESULTS:**

The results suggested that outpatients from the gastroenterology clinic were less dependent on alcohol, had suffered fewer consequences from alcohol and had fewer emotional and mental health problems than did the outpatients from the alcohol treatment service. In relation to their stages of change, the gastroenterology out-patients presented high precontemplation scores at the beginning of treatment while outpatients of alcohol treatment service showed higher scores in contemplation, action and maintenance.

**CONCLUSION:**

The medical treatment may be a reason for the temporary alcohol abstinence behavior among the gastroenterology outpatients.

## RESUMO

**CONTEXTO E OBJETIVO:**

Para alguns pacientes que desenvolveram doenças físicas relacionadas ao consumo de álcool, a abstinência pode oferecer a melhor chance de sobrevivência. O objetivo deste estudo foi de investigar a motivação para tratamento em dois grupos de consumidores de álcool: pacientes do ambulatório de gastroenterologia e do ambulatório especializado no tratamento para alcoolismo.

**TIPO DO ESTUDO E LOCAL:**

Corte transversal, no hospital-escola do serviço público federal.

**MÉTODOS:**

A amostragem estudada foi de 151 pacientes do ambulatório de gastroenterologia e 175 do ambulatório especializado. A entrevista foi conduzida nos ambulatórios na primeira consulta ao serviço e consistiu em uma seção com dados demográficos e escalas que avaliaram qualidade de vida, dependência de álcool, padrão de consumo alcoólico, motivação para tratamento e conseqüências decorrentes do beber.

**RESULTADOS:**

Os resultados sugeriram que os pacientes do ambulatório de gastroenterologia eram menos dependentes do álcool, sofriam menos as conseqüências relacionadas ao consumo de bebidas alcoólicas e possuíam menos problemas mentais e emocionais quando comprados com os pacientes do ambulatório para tratamento do alcoolismo. Em relação aos estágios de mudança, os pacientes da gastroenterologia apresentaram maiores escores na precontemplação no início do tratamento e os pacientes do ambulatório especializado apresentaram escores maiores em contemplação, ação e manutenção.

**CONCLUSÃO:**

O tratamento médico pode ser uma razão para a abstinência temporária de álcool para os pacientes do ambulatório de gastroenterologia.

## INTRODUCTION

Up to 80% of people with alcohol problems do not seek help from either specialist treatment services or self-help groups.^1^ However, untreated alcoholics incur general health care costs that are at least 100% higher than those for non-alcoholics, and this disparity may have existed for as long as 10 years prior to treatment entry.^2^ Most alcohol users will access either general medical services or social services during this time.^1,3^

In Brazil, a comprehensive national survey, performed in 107 large cities, indicated that 67.8% of the population had used alcohol at some point in their lives and, within this figure, 11.2% had had alcohol dependence. In the northern and northeastern regions of Brazil, these percentages reached 16%. In all regions there were more male than female alcohol users, in a ratio of 3:1.^4^ In the Brazilian Psychiatric Morbidity Survey, conducted in three major urban areas in Brazil using the diagnostic criteria of the Diagnostic and Statistical Manual of Mental Disorders, Third Edition (DSM-III), the prevalence of alcohol dependence was 15%.^5^

A survey undertaken at the same hospital as the current study used the Alcohol Uses Disorders Identification Test (AUDIT) to measure the frequency of alcohol misuse in a general public hospital and found that 22% of male inpatients and 3% of female inpatients scored positive. The highest prevalence was in the gastroenterology wards (27%). Of the 275 inpatients interviewed, 29% had a past history of alcohol consumption higher than the current level and, of these, 52% scored positive on AUDIT.^6^

For some patients who have developed significant alcohol-related physical complications, total abstinence from alcohol may offer the best chance of survival.^7^ However, if such patients underestimate the severity of their drinking problem or do not believe that their drinking behavior exacerbates their health problems, they may be unlikely to remain alcohol-free.

## OBJECTIVE

The aim of the current study was to assess outpatients from two different types of treatment service (a gastroenterology clinic and an alcohol treatment service) and compare their drinking and smoking behavior, quality of life, consequences of alcohol consumption and stages of motivational change, so as to discuss the implications for the type of intervention that is most appropriate.

## METHODS

### Design

This was a cross-sectional study.

### Setting

The study was undertaken at a general hospital. Two clinics were used: a gastrointestinal disease clinic and an alcohol treatment service. We considered it important to have outpatients in the sample who might display very different levels of motivation in relation to seeking help for their alcohol problem, hence the choice of the two settings.

### Sample

There were 151 outpatients interviewed in the gastroenterology clinic and 175 at the alcohol treatment service. All the outpatients were alcohol-dependent. The interviews were conducted in the outpatient clinics at the subjects’ first appointment, by one of three psychologist interviewers.

The inclusion criteria were as follows:

- At the alcohol treatment service: all outpatients who sought help for alcohol-related problems and who scored mild, moderate or severe alcohol dependence on the Alcohol Dependence Data Questionnaire (SADD).^8^
- At the gastrointestinal disease clinic: all At the gastrointestinal disease clinic: all outpatients were screened with the Portuguese version of AUDIT^6^ and those scoring positive (score ≥ 8) were interviewed using SADD. Thereafter, the same criteria were applied. During the 21-month study period, 336 outpatients came to the gastroenterology clinic, of whom 158 (47%) scored positive on AUDIT, but seven of them refused to take part in the study.

The most common illnesses diagnosed at the gastroenterology clinic were: 57% (n = 86) hepatopathy, 8% (n = 12) hepatitis, 6% (n = 9) pancreatitis, 14% (n = 21) no diagnosis and 15% (n = 23) others.

The exclusion criteria were: outpatients abusing substances other than alcohol, out-patients with gastrointestinal disease who were not alcohol-dependent and outpatients who presented high levels of intoxication during the interview. Women were also excluded from the study. As there were so few women presenting to either service, it was thought better to exclude them, as gender might have been a confounder in subsequent analyses.

### Main measurements

The interview consisted of the following:

- Sociodemographic data: age, schooling level, race, marital status, occupation and family income expressed as multiples of the Brazilian minimum salary (approximately equivalent to 70.00 United States dollars per month).
- The pattern of alcohol consumption was documented using the interview schedule developed for the World Health Organization/International Society for Biomedical Research on Alcoholism (WHO/ISBRA) Collaborative Study on State and Trait Markers in Alcoholism.^9^ The questions addressed the pattern of alcohol consumption, the quantity and frequency of use in the last 30 days and also the heaviest lifetime period of alcohol consumption.
- Short-Form Alcohol Dependence Data Questionnaire (SADD): derived from the original Attention Deficit Disorder (ADD) consisting of 15 items.^10^ The Brazilian Portuguese^8^ version measures the severity of alcohol dependence (mild, moderate and severe).
- Short-Form Health Survey (SF-36): to investigate quality of life through a general evaluation of health. This instrument was validated in Brazil by Cicconelli.^11^ The scale has 36 items which evaluate: physical functioning, role limitation due to physical problems, bodily pain, general health perceptions, vitality, social functioning, role limitation due to emotional problems, general mental health.
- The Drinker Inventory of Consequences (DrInc 2-L): this instrument (50 items) evaluates lifetime drink-related problems and was developed for the Michigan Assistive Technology Clearing House project (MATCH).^12^ It was translated and adapted to Brazilian Portuguese.^13^
- University of Rhode Island Change Assessment Scale (URICA): to investigate the stages of change: precontemplation, contemplation, action and maintenance. The questionnaire was translated and cross-culturally adapted into Portuguese, and back-translated into English. A Brazilian Portuguese version with Cronbach's alpha of between 0.63 and 0.79 was used.^13^

The model for the stages of change by Prochaska and DiClemente^14^ proposes a general and comprehensive explanation for understanding addictive behaviors, in relation to the way people change their behavior. A sequence of progressive and sequential stages are described (contemplation, precontemplation, action and maintenance), in which the motivation or intention to change can be considered through a “continuum”, without people necessarily moving through this continuum. The distinct stages of change in this sequence may lead towards the modification of the drinking habit, or not. In order to measure the stages of change, Prochaska and DiClemente developed URICA to include items marking the stages of change from their model.

g) The Stages of Change, Readiness and Treatment Eagerness Scale (SOCRATES): to investigate the readiness to change drinking behavior through recognition, ambivalence and taking steps. A Brazilian Portuguese version with Cronbach's alpha of between 0.74 and 0.89 was used. The questionnaire was translated and cross-culturally adapted into Portuguese, and back-translated into English. The confirmatory factor analysis showed that two correlated factors provided the best fit for the data.^15^ SOCRATES was originally developed for parallel measurement of the stages of change described by Prochaska and DiClemente, with item content specifically focused on problem drinking.

### Ethical considerations

The study was approved by the Medical Research Ethics Committee of Universi-dade Federal de São Paulo, Brazil, and is in accordance with the principles laid down in the Declaration of Helsinki (1964). All subjects signed a consent form prior to participating and were guaranteed anonymity and confidentiality.

### Statistical methods

The characteristics of the two samples of alcohol-dependent individuals were compared using the chi-squared test (χ^2^) for categorial data and Student's t test, for parametric variables that followed a normal distribution. Data that did not follow a normal distribution was analyzed by means of the non-parametric Mann-Whitney U test. Statistical significance was assigned if *p* were less than 0.05.

## RESULTS

*Sociodemographic data.* The demographic characteristics of the subjects according to the two group categories are presented in Table 1. The groups were similar with regard to race (white), marital status (married) and occupational status. The alcohol treatment service out-patients were in general better educated and younger than those from the gastric intestinal disease clinic.

**Table 1.** Sociodemographic data on alcohol-dependent outpatients who presented to an alcohol treatment service and to a gastrointestinal diseases clinic at a general hospital: percentage (number)

Sociodemographic data	Alcohol treatment service (n = 175)	Gastrointestinal disease clinic (n = 151)	p-value
**Age** (Mean ± SD)	41.31 (9.80)	47.60 (10.69)	0.001
**Level of schooling:**			
Illiterate	2.9 (5)	7.3 (11)	0.007
Basic education (8 years of schooling)	51.4 (90)	64.2 (97)	
High school education	29.7 (52)	19.9 (30)	
College/university	16.0 (28)	8.6 (13)	
**Race:**			
White	73.1 (128)	70.9 (107)	0.647
Non-white (black and mixed race)	26.9 (47)	29.1 (44)	
**Marital status:**			
Married	60.6 (106)	68.2 (103)	0.152
Unmarried	39.4 (69)	31.8 (48)	
Occupation:			
Blue-collar job	29.1 (51)	25.2 (38)	0.095
White-collar job	32.0 (56)	22.5 (34)	
Unemployed	30.3 (53)	40.4 (61)	
Other	8.6 (15)	11.9 (18)	
**Family income:**			
1 to 5 m.s.*	38.3 (67)	41.1 (62)	0.327
5 to 10 m.s.*	29.1 (51)	35.8 (54)	
10 to 20 m.s.*	17.1 (30)	13.9 (21)	
Up to 20 m.s.*	11.4 (20)	6.0 (9)	
Unknown	4.0 (7)	3.3 (5)	

* m.s. = minimum salary (1 minimum salary = 70.00 United States dollars per month at the time of data collection).

*Pattern of alcohol consumption and consequences of drinking.* There were marked differences in alcohol consumption. More outpatients from the alcohol treatment service scored higher on the SADD and more of them were rated as having severe alcohol dependence, while outpatients from the gastric clinic were more likely to have moderate dependence. Outpatients seen in the gastroenterology clinic tended to have had a moderate pattern of alcohol consumption over the last 30 days, while those seen in the alcohol treatment service were heavy drinkers. There was a longer time interval since outpatients had last consumed alcohol in the gastric clinic than in the alcohol treatment service.

There was no difference in the highest-ever level of reported alcohol consumption between the two groups, but the outpatients from the gastric clinic had drunk at this level for longer.

*Quality of life.* Outpatients from the alcohol treatment service had higher scores on the physical functioning, role limitation due to physical problems and vitality scales of SF-36, whilst gastroenterology outpatients scored higher on the role limitation due to emotional problems and general mental health sub-scales (Table 3).

**Table 2.** Comparison of severity of alcohol dependence and pattern of alcohol consumption, for alcohol-dependent outpatients presenting to an alcohol treatment service and a gastrointestinal diseases clinic: percentage (number), except where otherwise stated

		Alcohol treatment service (n = 175)	Gastrointestinal disease clinic (n = 151)	p-value
**Severity of alcohol dependence (SADD)**	Mild *	9.7 (17)	29.8 (45)	0.001
	Moderate *	30.3 (53)	39.1 (59)	
	Severe *	60 (105)	31.1 (47)	
**Pattern of alcohol consumption**	Total consumption in last 30 days *:			
	Moderate drinking (< 83 units per month)	25.1 (44)	79.5 (120)	0.001
	Heavy drinking (≥ 84 units per month)	74.9 (131)	20.5 (31)	
	Duration in weeks of present consumption: median	48	16	0.002
	(interquartile range) †	(8 - 144)	(8 - 32)	
	Days since last alcohol use:	3	38	0.001
	median (interquartile range) †	(1-14)	(11 -120)	
	Total monthly consumption during period of heaviest consumption: ‡			
	Moderate drinking * (< 83 units per month)	1.4 (2)	2.1 (3)	0.658
	Heavy drinking * (≥ 84 units per month)	98.6 (139)	97.9 (139)	
	Duration in weeks of heaviest period of consumption:	144	288	0.001
	median (interquartile range) †,‡	(63-336)	(96-681)	

* X^2^ = chi-squared;

† Z = Mann-Whitney;

‡ alcohol treatment service (n = 141) and gastrointestinal disease clinic (n = 142).

**Table 3.** Comparison of quality of life and drink-related problems between alcohol users from an alcohol treatment service and a gastrointestinal diseases clinic

	Scales	Subscales	Alcohol treatment service (n = 175)		Gastrointestinal disease clinic (n = 151)		t test	p-value
			Mean	SD	Mean	SD		
**Quality of life through a general evaluation of health**	SF-36	Physical functioning	79.64	19.91	65.39	25.99	5.599	0.001
		Role limitation due to physical problems	55.26	40.39	38.11	39.11	-3.878	0.0001
		Bodily pain	61.10	26.51	56.83	27.04	1.437	0.152
		General health perceptions	61.70	21.67	59.34	20.91	0.994	0.321
		Vitality	58.43	23.48	55.65	26.42	-1.004	0.023
		Social functioning	58.2	28.96	60.59	32.08	0.699	0.485
		Role limitation due to emotional problems	41.06	39.56	51.80	40.29	2.423	0.016
		General mental health	55.24	24.60	61.42	23.79	-2.297	0.022
**Drink-related problems**	DrInc 2-L	Total score	32	9	26	11	5.943	0.001

SF-36 = Short Form Health Survey-36; the SF-36 scores are on a scale where 100 is the best quality of life and 0 is the worst; DrInc 2-L = Drinker Inventory of Lifetime Consequences; SD = standard deviation.

*Drink-related problems.* The outpatients from the alcohol clinic scored higher on the DrInc 2-L questionnaire, thus suggesting that they had suffered more alcohol-related problems in their lifetimes than had the outpatients in the gastric clinic (Table 3).

*Motivation to treatment.* With regard to the stages of change, significant differences were found on all the subscales of URICA, with the alcohol treatment service group scoring higher in the contemplation, action and maintenance domains and the gastroenterology outpatients scoring highest on the precontemplation scale (Table 4). In SOCRATES, the outpatients from the alcohol treatment service scored higher on recognition and ambivalence, but there were no differences between the two samples in the scores for taking steps.

**Table 4.** Comparison between alcohol-dependent outpatients from an alcohol treatment service and a gastrointestinal diseases clinic using the Stages of Change, Readiness and Treatment Eagerness Scale (SOCRATES) and the University of Rhode Island Change Assessment Scale (URICA)

Scales	Subscales	Alcohol service (n = 175)		Gastrointestinal disease clinic (n = 151)		t test	p-value
		Mean	SD	Mean	SD		
**URICA**	Precontemplation	18.93	5.26	20.55	4.66	-2.923	0.004
	Contemplation	29.78	2.66	28.44	2.68	4.498	0.001
	Action	28.49	3.32	27.63	3.19	2.365	0.019
**SOCRATES**	Maintenance	22.92	4.97	21.29	4.74	3.012	0.003
	AMREC	35.61	5.24	30.21	7.03	7.921	0.001
	Taking steps	26.71	3.94	26.89	4.12	-0.402	0.688

AMREC = Ambivalence and recognition; SD = standard deviation.

## DISCUSSION

In this study we have compared two groups of alcohol-dependent outpatients: one from a specialist alcohol treatment service and one from a gastrointestinal diseases clinic. Marked differences were found in the characteristics of these two populations. The outpatients from the alcohol treatment service were younger, but more severely dependent on alcohol and more likely to be current heavy drinkers. Despite their relative youth, they had suffered more alcohol-related problems. The outpatients from the alcohol service had suffered more emotional and mental health problems but fewer physical consequences.

In terms of their motivational state, the outpatients from the alcohol clinic were more likely to believe that they had a drink problem and needed to seek help to change their drinking behavior. The outpatients from the gastrointestinal clinic were older and had been drinking for longer. Although in the past they had been drinking at a level similar to that of the outpatients from the alcohol clinic, they were now more likely to be only moderately dependent, to have recently cut down their consumption or to have stopped drinking during the last month. Even though they had been drinking for longer, they had accrued fewer lifetime alcohol-related problems and had fewer role limitations due to emotional and general health problems. Despite this, their cognitive beliefs seemed to be lagging behind and they were more likely to be in a precontemplative stage as far as changing their drinking behavior was concerned.

Since the outpatients from the alcohol treatment clinic were voluntarily seeking help for their problems, it is not surprising that they scored higher on the questionnaires measuring contemplation, action and maintenance. What is surprising is why the gastrointestinal disease outpatients, who had serious physical complications requiring medical treatment and had already changed both their drinking and smoking behavior, should score so high on the precontemplation scale but low on the action and maintenance scales. It could be argued that, as the gastrointestinal disease outpatients had already changed their drinking behavior, they did not see the need to take further action or seek help and, for this reason, they scored high on the precontemplation stage. However, if this were the case, it would be expected that they would score high on the action and maintenance subscales, which they did not. It seems as if this group of outpatients are just stopping alcohol consumption while undergoing treatment and/or because of gastrointestinal disease symptoms. It is important to note that drinking behavior was strongly advised against by the gastroenterologists in this study. The treatment may be the reason for their temporary alcohol abstinence, although it may not be able to change the behavior. Another reason for this may be that outpatients with alcohol-related liver disease are generally less dependent on alcohol.^16,17^ However, there is evidence that drinkers who have developed liver disease have limited insight into the relationship between their behavior and their health status,^18^ and they do not differ significantly from patients with non-alcohol related liver diseases in the level of awareness of the severity of their disease.^19^ Many people with alcohol dependence who suffer from secondary illnesses will not accept onward referrals to alcohol treatment programs.^20,21^ Furthermore, healthcare professionals working in medical services may not be skilled at recognizing or treating alcohol misuse.^22^ Therefore, it may be more appropriate for any intervention undertaken with this patient group to take place in the gastroenterology clinic itself, by trained specialist alcohol liaison workers.^23,24^

The implications of these findings in relation to the type of treatment intervention that is appropriate to each patient group are important. Outpatients presenting to alcohol treatment services are more likely to be severely dependent drinkers and to be still drinking heavily when they come. Treatment should be aimed at enabling these outpatients to take the first step towards reducing their alcohol consumption or stopping altogether. At the time of presentation to services, they are likely to be motivated to change and receptive to cognitive behavioral approaches for facilitating such change.^25^ Treatment services need to be responsive to this state of readiness.

On the other hand, outpatients presenting to gastroenterology clinics with physical complications of alcohol misuse are more likely to have already taken steps to reduce or stop their alcohol consumption (a long time interval since such outpatients had last consumed alcohol), but are less likely to be receptive to the kind of interventions offered by specialist alcohol treatment services. The initial aims of treatment need to be more focused on engaging these outpatients with treatment services, thereby enabling them to see the link between their alcohol consumption and physical health problems. This will support the changes that they have already made and teach them the skills to reduce their risk of relapse back into alcohol misuse.^26^

## CONCLUSION

The outpatients from the gastrointestinal disease clinic showed low motivation to change the drinking behavior. The treatment may be a reason for their temporary alcohol abstinence, because the outpatients scored high on the precontemplation scale but low on the action and maintenance scales. It seems as if this group of outpatients are just stopping alcohol consumption while undergoing treatment and/or because of gastrointestinal disease symptoms. Further studies will be important, because there is little information about this subject.
